# A Novel High Recognition Rate Defect Inspection Method for Carbon Fiber Plain-Woven Prepreg Based on Image Texture Feature Compression

**DOI:** 10.3390/polym14091855

**Published:** 2022-04-30

**Authors:** Lun Li, Yiqi Wang, Jialiang Qi, Shenglei Xiao, Hang Gao

**Affiliations:** Key Laboratory for Precision and Non-Traditional Machining Technology of Ministry of Education, School of Mechanical Engineering, Dalian University of Technology, Dalian 116024, China; li_lun@mail.dlut.edu.cn (L.L.); wangyiqi@dlut.edu.cn (Y.W.); qijialiang@mail.dlut.edu.cn (J.Q.); gaohang@dlut.edu.cn (H.G.)

**Keywords:** plain-woven prepreg, defect inspection, texture feature compression, k-means algorithm

## Abstract

Carbon fiber plain-woven prepreg is one of the basic materials in the field of composite material design and manufacturing, in which defect identification is an important and easily neglected part of testing. Here, a novel high recognition rate inspection method for carbon fiber plain-woven prepregs is proposed for inspecting bubble and wrinkle defects based on image texture feature compression. The proposed method attempts to divide the image into non-overlapping block lattices as texture primitives and compress them into a binary feature matrix. Texture features are extracted using a gray level co-occurrence matrix. The defect types are further defined according to texture features by k-means clustering. The performance is evaluated in some existing computer vision and machine learning methods based on fiber recognition. By comparing the result, an overall recognition rate of 0.944 is achieved, which is competitive with the state-of-the-arts.

## 1. Introduction

Carbon fiber reinforced plastics are widely used in aerospace due to their good fatigue and corrosion resistance coupled with high strength-to-weight and stiffness-to-weight ratios [1]. The predominant material when it comes to composites in structural aircraft components is pre-impregnated carbon fibers [2]. The preparation process of composites means that it is easy to produce bubble and wrinkle defects, and timely inspection of these defects can help improve both the performance of composites and their subsequent service performance.

The inspection methods for examining wrinkle and bubble defects in carbon fiber prepreg are not like the common non-destructive testing methods for composite materials, which are similar to surface texture inspection methods. Unidirectional prepreg and woven prepreg are the most popular raw materials for fabricating composite materials. Unidirectional prepreg is the most commonly utilized material thanks to automatic manufacturing processes such as automated tape laying and automatic fiber placement. With improvement in the degree of automation, defect inspection technologies for unidirectional prepreg have gained popularity as well; these include ultrasonic testing [3], radiographic testing [4], and thermal imaging testing [5]. The structure of woven prepreg is more complicated than that of unidirectional prepreg, and the quality inspection of its preparation process is more difficult. At present, defect inspection for carbon fiber woven prepreg relies heavily on manual inspection, which has the disadvantages of low detection efficiency dependence on the experience of the inspector. Moreover, due to the complex structure of carbon fiber woven prepreg, when defect inspection technologies for unidirectional prepreg are used to examine defects in carbon fiber woven prepreg, the recognition rate of the inspection technologies used with unidirectional prepreg cannot meet the demands of examining carbon fiber woven prepreg. Therefore, it is particularly important to propose a new defect inspection method with a high recognition rate for use with carbon fiber woven prepreg, in particular to allow for online inspection during future automated manufacturing processes.

From a woven fabric texture feature point of view, there are typically four classes [6,7]: structural analysis (SA) [8,9], spectral methods [10,11,12], model-based methods [13,14,15], and statistics-based classification approaches [16,17,18]. Among these, SA assumes that the surface texture is generated by following a placement rule and that defect-frees and defects are respectively composed of overlapped texture primitives and nonoverlapped texture primitives. Therefore, SA is one of the most suitable methods for defect inspection of woven prepreg; its core texture primitives are defined differently in the literature. Bodnarova et al. [10] introduced the definition of texture primitive as texture blobs surrounded by rectangular regions to form an overlapping binarized grid. Ng tested the performance of the valley-emphasis method on common defect detection applications [19]. Jia and Liang [20] proposed a texture blob location-based method which does not directly assume that it conforms to a rigid grid, rather inferring the placement rule dynamically. As stated in [6,7], the requirement of the texture pattern is regular; thus, the locations of defects can be identified through structural analysis.

From the perspective of training strategy, woven prepreg inspection methods are divided into two classes based on either supervised or unsupervised training. Unlike unsupervised machine learning methods, woven prepregs may have unpredictable defect forms in different production processes; thus, manufacturing inspection methods are different from traditional machine learning methods, e.g., image decomposition (ID) [21], motif-based (MB) methods [22], Bollinger bands (BB) [23], regular bands (RB) [24], Elo rating (ER) methods [25], and wavelet-pre-processed golden image subtraction (WGIS) [26]. The ID method inspects defects using the integrating the image decomposition method, which allows for the possibility of removing repeated texture primitives completely and removing segment defects directly [27,28,29]. Another special method, MB [22], is rarely mentioned in the literature on plain-woven fabric inspection; its distinguishing characteristic is the preprocessing step of lattice segmentation. The lattice segmentation method [30] divides a plain-woven fabric image into non-overlapping block lattices which consist of the same textures. The block lattice is similar to the texture primitives in SA, and the lattice segmentation method derives a unified placement rule for images of the same woven fabric patterns which share roughly the same texture for segmented block lattices. The two categories (defect-frees and defects) generalize the categorization of motif-based and non-motif-based methods.

The vision testing and lattice segmentation methods are exploited here to develop a novel plain-woven fabric image analysis method including texture feature compression, which is proposed for synthetically analyzing the texture patterns of carbon fiber plain-woven prepreg and classify them into defect-free, bubble defect, and wrinkle defect classes. Texture features are extracted using a gray level co-occurrence matrix (GLCM). The types of defects are further defined according to their texture features. Finally, k-means clustering (KMC) is used to confirm the types of defects, then detect the quality of prepreg for effective processing. This method can accurately extract the texture feature information of carbon fiber plain-woven prepreg, making inspection results more reliable and realistic.

## 2. Methodology

### 2.1. Image Preprocessing

Prepreg images acquired from via digital cameras are embedded with errors such as noise, fickle shadows, and illumination changes; these can appear similar to defective objects caused in the manufacturing process and affect image quality. Thus, image preprocessing can dampen the bad effects caused by such errors, which is vital in feature extraction [31,32]. Three kinds of preprocessing methods were used here to enhance captured images as well as to determine crossover points or floats (the technical details of these calculations can be found in Appendix A). The main preprocessing methods used were presented as follows.

Step 1. Histogram equalization: The plain-woven texture of the images was equalized by cumulative distribution function (CDF), which is helpful for enhancing their contrast.

Step 2. Gray-level morphology: The bottom-hat image was subtracted from the sum of the original and top-hat images in order to maximize the contrast between the objects and the gaps and distinguish them from each other.

Step 3. Steerable filters: Image edges were obtained by deconvolving the image with templates generated in different directions. Then, Hough transform was used to strengthen the weft yarn in the image, and the image was corrected according to the inclination angle. Meanwhile, performance evaluation metrics were utilized to compare the results of the vertical and horizontal filters.

### 2.2. Image Compression

The woven pattern of carbon fiber plain-woven prepreg has different describable symmetry features depending on the warp and weft yarns. Based on this symmetrical woven pattern, a new image compression algorithm which can remove noise data, retain texture features, and compress and simplify the original image data is proposed here. At the same time, the texture primitive of plain-woven patterns can be extracted from defect-free images. The texture primitive is a unit that can form a whole pattern via simple translation without rotation [33]. In addition, it is a marker for recognizing the woven pattern of carbon fiber plain-woven prepreg.

**Figure 1:**
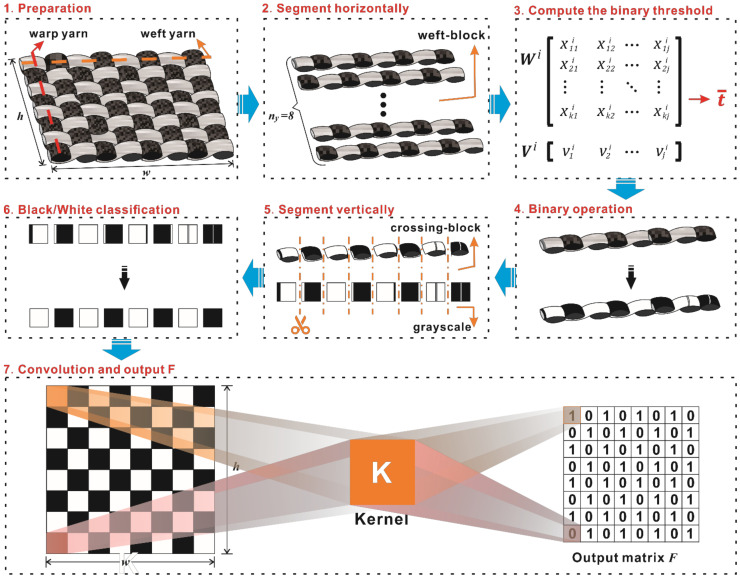
The schematic of texture features in the compression process.

As shown in Figure 1, taking a defect-free carbon fiber plain-woven prepreg as an example, the image compression process here was carried out as follows.

Step 1. Preparation: The original matrix ***O****_org_* can be obtained from a preprocessing image, and the pixel width *w* and pixel height *h* can be determined as well. In the process of capturing the surface image of each prepreg, we determined the focal length, FOV, and working distance of the camera and the morphological structure of the prepreg did not change. Therefore, the numbers of warps and wefts in each image could be determined, and are defined here as *n_x_* and *n_y_*, respectively.

(1) $$O_{org}=\left[\begin{array}{ccc} o_{11} & \cdots & o_{1j}\\ \vdots & \ddots & \vdots \\ o_{k1} & \cdots & o_{kj} \end{array}\right],\;\;j=1,2\cdots ,w\;\;;\;k=1,2\cdots ,h$$

Step 2. Segment horizontally: the original matrix, ***O****_org_*, is segmented horizontally according to the value of the weft, *n_y_*. As a result, *n_y_* weft-block images corresponding to the matrix of *n_y_* weft-block images can be obtained as shown in Equation (2). The number of rows and columns in each matrix is respectively *r_h_* and *w*, where *r_h_* = *h*/*n_y_* and *x* represents the gray value in the weft-block image. Then, the weft-block and original matrices can be expressed as shown in Equations (3) and (4):

(2) $$x_{j}^{i}={\left[\begin{array}{ccc} x_{1j}^{i} & \cdots & x_{kj}^{i} \end{array}\right]}^{T},\;i=1,2\cdots ,n_{y}\;\;\;;\;j=1,2\cdots ,w\;\;;\;k=1,2\cdots ,r_{h}$$

(3) $$W^{i}=\left[\begin{array}{ccc} x_{1}^{i} & \cdots & x_{j}^{i} \end{array}\right]=\left[\begin{array}{ccc} x_{11}^{i} & \cdots & x_{1j}^{i}\\ \vdots & \ddots & \vdots \\ x_{k1}^{i} & \cdots & x_{kj}^{i} \end{array}\right]$$

(4) $$O_{org}={\left[W^{1}\;,\;W^{2}\;\cdots \;,W^{i}\right]}^{T}$$

Step 3. Compute the binary threshold value, $\bar{t}$; the elements (gray value) of each column in the matrix ***W**^i^* are added up together and then multiplied by a correction coefficient, λ, as shown in Equation (5). The weft-block matrix ***W**^i^* becomes a new matrix, ***V**^i^*, as shown in Equation (6).

(5) $$v_{j}^{i}=\lambda \times \sum_{k=1}^{r_{h}}x_{kj}^{i}\;,\;\;\;i=1,2\cdots ,n_{y}\;\;\;;\;j=1,2\cdots ,w$$

(6) $$V^{i}=\left[\begin{array}{ccc} v_{1}^{i}\;, & v_{2}^{i}\;, & \cdots \;\;, \end{array}\;\;\;\;v_{j}^{i}\right]$$

Afterwards, the element sequence of ***V**^i^* is used as the x-coordinate and the element value as the y-coordinate to draw the grayscale transformation graph; in other words, the variations in the element sequence of ***V**^i^* in terms of grayscale values are plotted against the number of pixels. A sub-threshold value, *t_i_*, can be easily obtained from the grayscale transformation graph. Similarly, the binary threshold value, $\bar{t}$, can be obtained by taking the average of the corresponding thresholds of each obtained ***V**^i^*, as expressed in Equation (7):

(7) $$\bar{t}=\sum t_{i}/i\;,\;\;i=1,2\cdots ,n_{y}$$

Step 4. Binary operation: the binary threshold, $\bar{t}$, is used to divide the element value of each column vector $x_{j}^{i}$ into binary data (0 or *α*) in matrix ***W**^i^*, as shown in Equation (8). Thus, the original matrix, ***O****_org_*, and the warp-block matrix ***W**^i^* become the binary matrices ***O****_bnr_* and ***W**^i^_bnr_*, respectively.

(8) $$x_{j}^{i}=\left\{\begin{array}{c} 0,\;\;v_{j}^{i}<\bar{t}\\ \alpha ,\;\;v_{j}^{i}\geq \bar{t} \end{array}\right.$$

Generally, the pixel values 0 and 255 represent black and white in a grayscale image. For a good data visualization effect, the pixel value *α* is usually defined as 255.

Step 5. Segment vertically: the binary matrix ***W**^i^_bnr_* is segmented vertically according to the value of the warp, *n_x_*. As a result, *n_x_* crossing-block images and the corresponding *n_x_* crossing-block matrix ***C^il^*** in each ***W**^i^_bnr_* can be obtained as shown in Equation (9), with ***C**^il^* being a binary matrix. The number of rows and columns of each matrix ***C**^il^* is *r_h_* and *r_w_,* respectively, where *r_h_* = *h*/*n_y_*, *r_w_* = *w*/*n_x_*. Then, the binary warp-block and binary original matrices can be expressed as shown in Equations (10) and (11):

(9) $$C^{il}=\left[\begin{array}{ccc} x_{1}^{il} & \cdots & x_{j}^{il} \end{array}\right]=\left[\begin{array}{ccc} x_{11}^{il} & \cdots & x_{1j}^{il}\\ \vdots & \ddots & \vdots \\ x_{k1}^{il} & \cdots & x_{kj}^{il} \end{array}\right],\;\;l=1,2\cdots ,n_{x}\;\;\;;\;j=1,2\cdots ,r_{w}\;\;;\;k=1,2\cdots ,r_{h}$$

(10) $$W^{i}{}_{bnr}=\left[\begin{array}{ccc} C^{i1} & \cdots & C^{il} \end{array}\right]$$

(11) $$O_{bnr}={\left[W^{1}{}_{bnr}\;,\;W^{2}{}_{bnr}\;\cdots \;,W^{i}{}_{bnr}\right]}^{T}$$

Step 6. Black/White classification: the binary proportions of each element are calculated for the crossing-block matrix ***C**^il^*; the color with a smaller proportion will be replaced by the color with a larger proportion. In terms of a grayscale image, the matrix ***C**^il^* is constituted by either a black block or a white block. A new matrix, ***O****_cls_*, can be obtained by updating the values of the elements of ***O****_bnr_*.

Step 7. Extract the output matrix ***F***: Convolution operations are performed between ***O****_cls_* and convolution kernel ***K***. The kernel ***K*** is an *r_w_* × *r_h_* matrix in which the values of all elements are the same as *ζ* calculating by Equation (12); the horizontal step is *r_w_* and the vertical step is *r_h_*. The output matrix ***F*** can be obtained from the following Equation (13):

(12) $$\zeta ={\left(r_{w}\times r_{h}\times \alpha \right)}^{-1}$$

(13) $$F=O_{cls}⨂K$$

where ⊗ is the convolution operator.

As a result, the output matrix ***F*** is obtained, in which the number of rows is equal to the number of weft yarns and the number of columns is equal to the number of warp yarns; it is a binary matrix that the element value is either zero or one. Thus, the texture feature of a defect-free carbon fiber plain-woven prepreg is clearly mapped by matrix ***F*** with the least amount of data.

### 2.3. Texture Feature Extraction

The GLCM is a texture feature extraction method based on gray-level spatial dependence. Each element in the matrix represents the occurrence of a grayscale combination. Assuming that *f*(*x*, *y*) is a two-dimensional digital image with a size of *M* × *N* and a gray level of *h*, the different pixel spacing modeling of GLCM with a certain spatial relationship is expressed as Equation (14) [34]:

(14) $$P\left(i,j\right)=\#\left\{\left(x_{1},y_{1}\right),\left(x_{2},y_{2}\right)\in M\times N\left|f\left(x_{1},y_{1}\right)\right.=i,f\left(x_{2},y_{2}\right)=j\right\}$$

where *#* denotes the number of elements in the set, *d* is the distance between (*x*_1_, *y*_1_) and (*x_2_*, *y_2_*), and ***θ*** is the angle between the vector and the axis of the coordinate.

**Figure 2:**
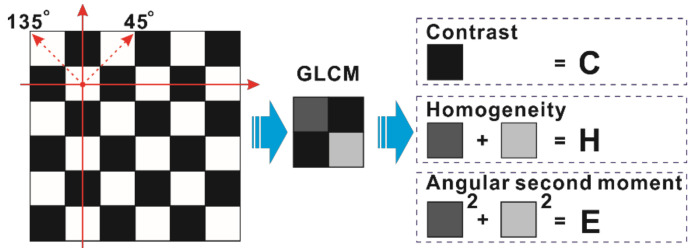
The schematic of three defect-free fiber texture feature extractions.

In general, *d* selects 1~8, while θ selects 0°, 45°, 90°, and 135°. According to the output matrix ***F*** provided by the texture feature compression algorithm, only one kind of the texture feature of weft and warp yarns can be extracted in a case where the angle of *θ* is 45° or 135°, as shown in Figure 2. Thus, the values of θ with 45° and 135° can be selected in order to improve the contrast of the feature data, thereby facilitating the detection of defect and defect-free images. The co-occurrence matrices of the two conditions are tabulated in Table 1.

Therefore, texture features can be represented by the values of contrast and homogeneity along with the angular second moment [35,36]. These three secondary statistics can reflect the textural features of carbon fiber plain-woven prepreg.

The schematic for defect-free fiber texture feature extraction is shown in Figure 2; the different colors of the square sections represent the different values of different textural features. The value of the white section is 1, the value of the black section is 0, and the values of dark gray and light gray sections are equal to the calculation results. Taking the defect-free image as an example, whether *θ* is 45° or 135° the size of a matrix is 2 × 2 and one diagonal value is 0. Therefore, the contrast, homogeneity, and angular second moment can be easily obtained using basic arithmetic (the specific operation is shown in Appendix A).

### 2.4. Defect Inspection

Bubble and wrinkle defects are two kinds of typical defects in prepreg during the laying up process. The KMC algorithm was adopted to inspect the textural features in defect-free, winkle defect, and bubble defect cases. By calculating the center of clustering for each condition, the KMC algorithm looked for the best way of grouping images with a minimal value of the mean similarity [37,38]. The accuracy of the KMC algorithm can be improved and verified using training and validation sets with images for the three conditions. Moreover, the KMC algorithm can consolidate the defined defect-type partition [39].

## 3. Materials and Experiments

**Figure 3:**
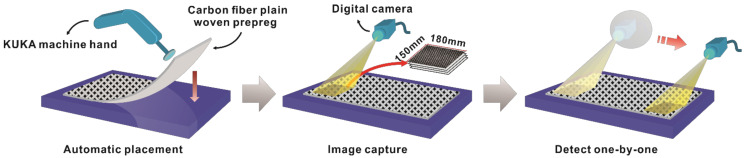
The process of defect inspection for carbon fiber plain-woven prepreg.

**Figure 4:**
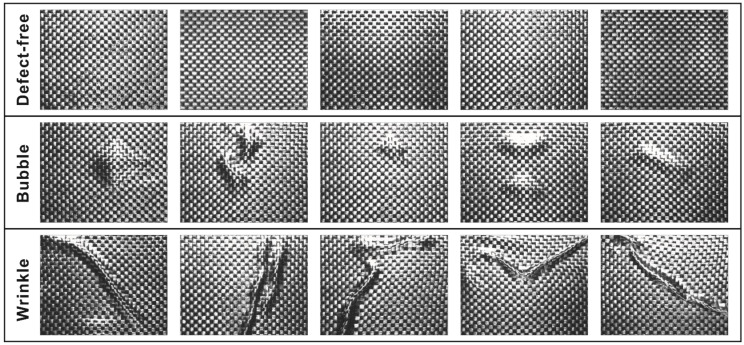
The original images of carbon fiber plain-woven prepreg with and without defects, captured by digital camera.

Carbon fiber plain-woven prepreg (WP-3011, Guangwei Composite Material Co., Ltd, Weihai, China) was used for this study. The prepreg was laid on the mold with assistance from a machine hand (KRC4, KUKA Robotics Co., Ltd, Shanghai, China). Then, images were captured using a digital camera (MV-CH050-10UM, HIKROBOT Co., Ltd, Hangzhou, China). The camera captured images of other areas by coordinate movement and stored them in the computer. The computer configuration used was a Windows 7 64x operating system and Inter Core i7-8565U (1.8 GHz) CPU with 8 GB running memory. The process of defect inspection for carbon fiber plain-woven prepreg is shown in Figure 3. The working distance of the camera was 450 mm. Defect sizes were no more than 150 mm × 180 mm, and not smaller than the size of the texture primitive. Representative original images of the defect-free, bubble defect, and wrinkle defect samples are shown in Figure 4.

As a result, 1200 prepreg surface images (200 defect-free images, 500 images with bubbles, and 500 images with wrinkles) were collected as the total dataset and used for improving the proposed plain-woven prepreg defect automatic inspection method.

**Figure 5:**
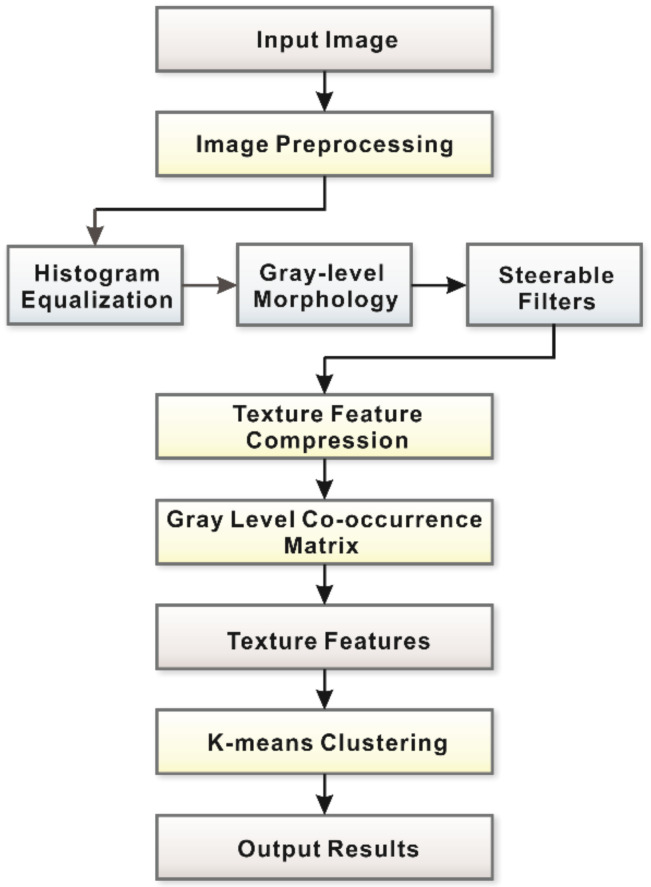
The flowchart of the proposed defect automatic inspection method.

Python 3.7 and OpenCV module were used for the development of the proposed defect automatic inspection method; a flowchart is shown in Figure 5. The enhanced grayscale images of the prepreg were obtained using three preprocessing algorithms. A texture feature compression algorithm was used to compress and simplify the preprocessed images. Then, the texture features of the compressed output matrix ***F***, consisting of contrast, homogeneity, and the angular second moment, were extracted by the GLCM algorithm (1-pixel and 45°, 135° directions). The mean value of two directions was utilized as the texture feature. These three features formed a three-element array as the input of the KMC algorithm. In the end, the results of clustering recognition were obtained.

## 4. Results and Discussion

**Figure 6:**
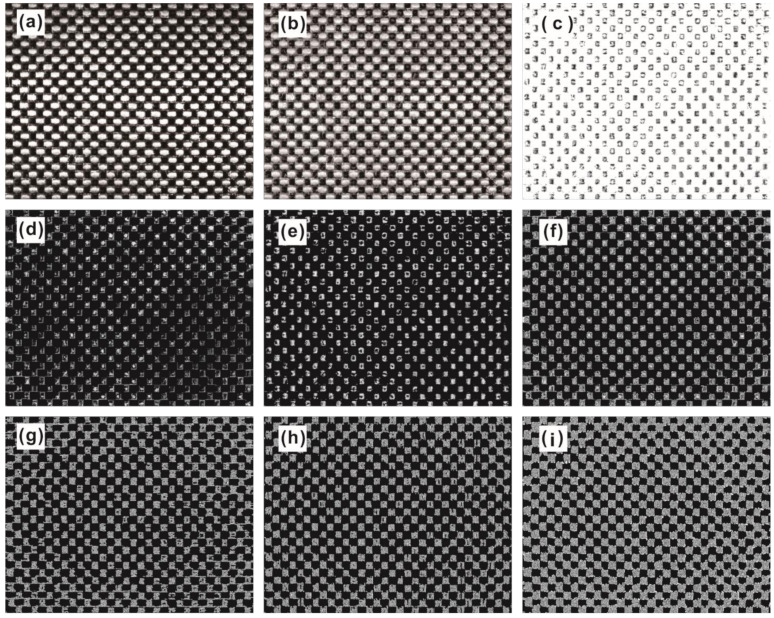
Image preprocessing steps: (**a**) original grayscale image; (**b**) histogram equalization; (**c**) box filtering; (**d**) top-hat transform; (**e**) bottom-hat transform; (**f**) gray-level morphology; (**g**) horizontal steerable filter; (**h**) vertical steerable filter; and (**i**) Gaussian filter.

As shown in Figure 6, a series of preprocessed defect-free images were used to describe the image preprocessing steps. The original grayscale image is shown in Figure 6a. To enhance the contrast in the original grayscale image, the plain-woven texture of the image was equalized by CDF, as shown in Figure 6b. Then, the histogram-equalized image was filtered through a box filtering function to reduce noise, as shown in Figure 6c. After that, a top-hat transform (as shown in Figure 6d) and bottom-hat transform (as shown in Figure 6e) were applied to the filtered image to obtain the gray-level morphology, as shown in Figure 6f. The gray-level morphology algorithm allowed us minimize the effects of tensile fiber on fiber texture extraction. Finally, a steerable filtering algorithm, which included a horizontal steerable filter (as shown in Figure 6g) and a vertical steerable filter (as shown in Figure 6h), was used to obtain the clear edges of each yarn, with the templates generated in different directions by the deconvolving method. With this comparison, the vertical steerable image was selected for the Gaussian filter to complete the whole image preprocessing step, as shown in Figure 6i.

**Figure 7:**
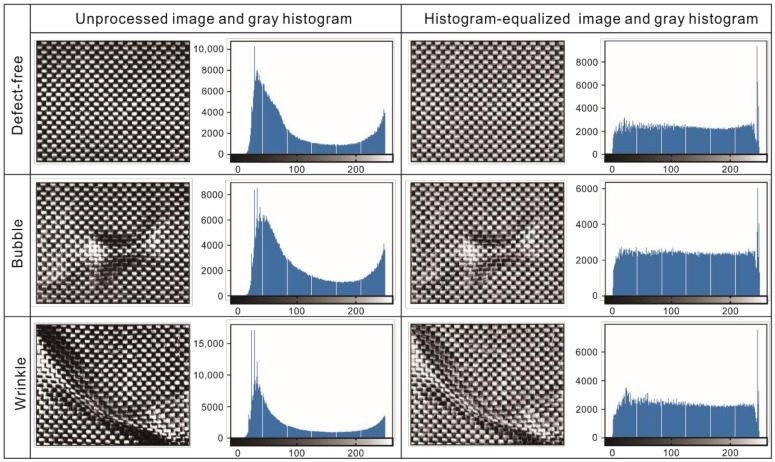
Three kinds of grayscale images (defect-free, bubble, and wrinkle) and their gray histograms with/without histogram equalization.

The comparisons between the original gray and the histogram-equalized images and histograms in three conditions (defect-free, bubble defect, and wrinkle defect) are shown in Figure 7. By comparing the images, it can easily be seen that the histogram equalization equalizes the brightness of the original grayscale image caused by the reflective resin on the prepreg surface and improves the clarity of the plain-woven texture. By comparing the histograms, it can be seen that a uniform distribution of the gray level was obtained, which is helpful for extraction of texture features.

**Figure 8:**
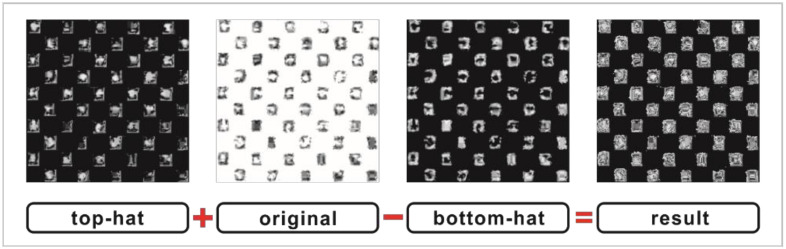
Gray-level morphological operation processing.

The gray-level morphological operation processing is shown in Figure 8. An area of 10 (warp yarns) × 10 (weft yarns) was intercepted for image observation. The weave points of warp and weft yarns in the image were regarded as mountains and valleys. The contrast was improved by minimizing the number of valleys. The top-hat and bottom-hat images contained the mountains and the valleys of the yarns, respectively. Thus, gray-level morphological operation processing was carried out by adding the top-hat operated image to the original grayscale image and then subtracting the bottom-hat operated image from the sum. Following this gray-level morphology process, the gray-level morphological image was obtained, which was similar to the ideal image.

**Figure 9:**
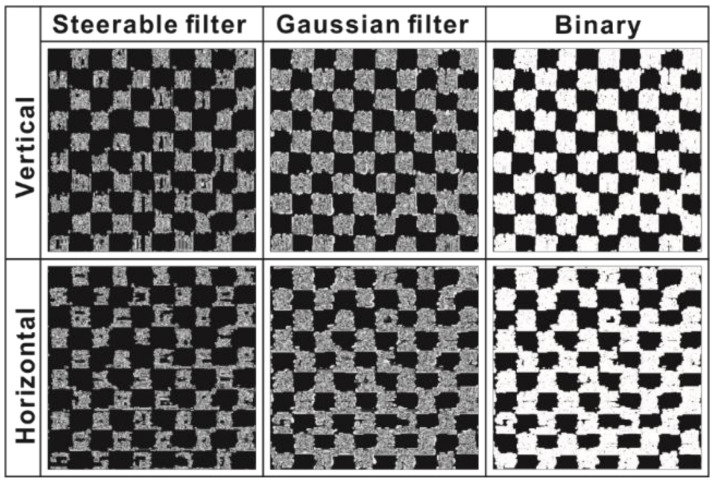
Comparison of the results of the vertical and horizontal steerable filters, Gaussian filter, and binary operation.

A steerable filter was adopted to filter the gray-level morphological image in order to emphasize the borders of warp and weft yarns in the final preprocessing step. The effects of vertical filtering and horizontal filtering are compared in Figure 9. The performance evaluation metrics for the vertical and horizontal steerable filtered images are shown in Table 2. From Table 2, it can be seen that (i) the ACC values of the vertical steerable filter are much higher than those of the horizontal steerable filter; (ii) the TPR values of the horizontal steerable filter in the full-size image are very low, specifically, 41.64% below the maximum value; and (iii) the FPR values of the vertical steerable filters are much lower than those of the horizontal steerable filters. Therefore, the vertical steerable filtered images were selected to improve recognition accuracy before the final preprocessing step.

**Figure 10:**
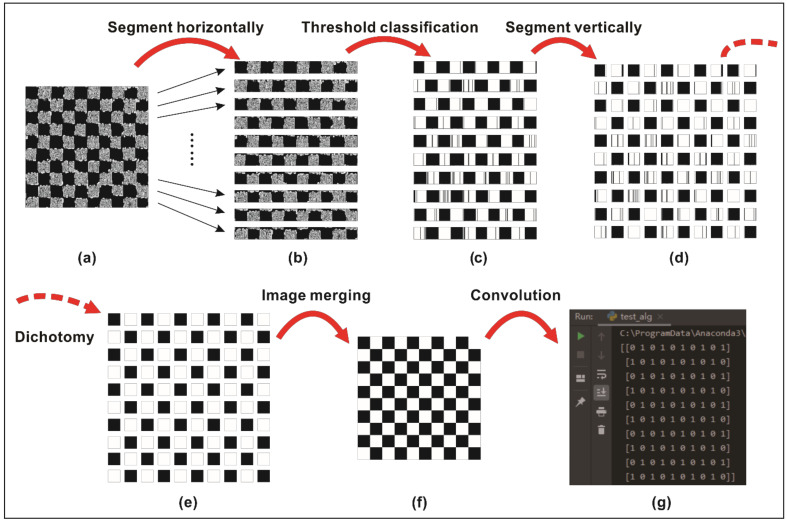
The image compression process: (**a**) preprocessed image; (**b**) grayscale sub-image arrays of ten weft yarns; (**c**) binary sub-image arrays of ten weft yarns; (**d**) preliminary texture pattern; (**e**) preliminary texture pattern processed by dichotomy; (**f**) final texture pattern; and (**g**) the output matrix, ***F***.

**Figure 11:**
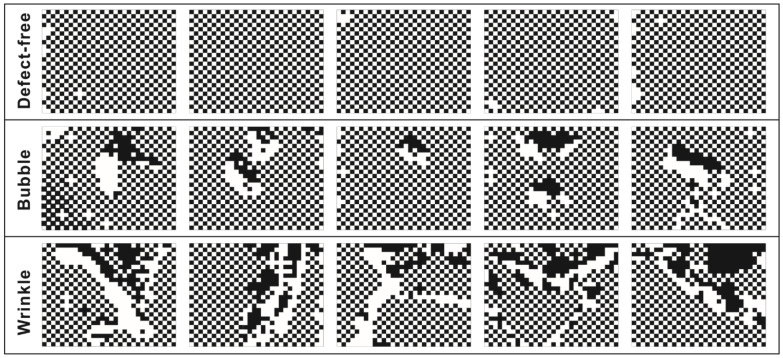
The texture patterns of the original images of carbon fiber plain-woven prepreg with and without defects, corresponding to Figure 4.

An image compression algorithm was proposed to compress and simplify the preprocessed image data while retaining the texture features for further analysis; the image compression process is shown in Figure 10. A preprocessed image (shown in Figure 10a) was segmented horizontally (shown in Figure 10b) based on the number of weft yarns, then used to compute the binary threshold value for threshold classification (shown in Figure 10c). The binary sub-image arrays were then segmented vertically based on the number of warp yarns to obtain crossing-block image arrays as the preliminary texture pattern, which are shown in Figure 10d. Then, a dichotomy operation was carried out for the Black/White classification process, as shown in Figure 10e. After that, the crossing-block image arrays were merged to obtain the final texture pattern, as shown in Figure 10f. Using a convolution operation, the final texture pattern was converted into the output matrix, ***F*** (obtained by Python and shown in Figure 10g), which was used as a standard defect-free template for further analysis. Following the same image compression process, the texture patterns of the original images of carbon fiber plain-woven prepreg with and without defects (corresponding to Figure 4) were obtained, and are shown in Figure 11.

Texture features were extracted based on the GLCM method. The mean values of the texture features are listed in Table 3. Three texture feature parameters, namely, the contrast and homogeneity along with the angular second moment, made up a three-dimensional array which was used to present the texture features.

**Figure 12:**
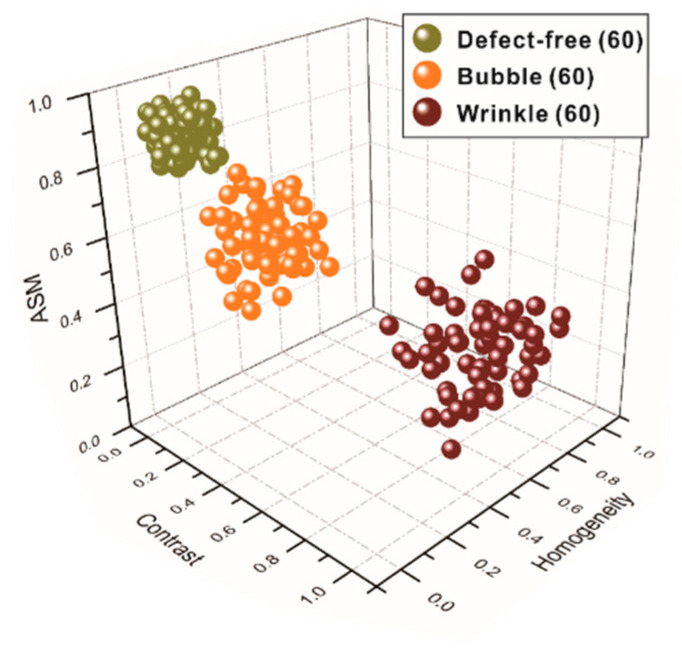
Plot of KMC algorithm classification for the three conditions.

The KMC algorithm classified 180 samples by three conditions, defect-free (60 samples), with bubble defect (60 samples), and with wrinkle defect (60 samples), by recognizing different representative defect-free, bubble, and wrinkle texture features of carbon fiber plain-woven prepreg samples randomly selected from the 1200 total samples. The results were plotted as a three-dimensional scatter plot, shown in Figure 12. Each texture feature of these three conditions has distinguishable differences, which are defined according to their locations in the plot.

**Figure 13:**
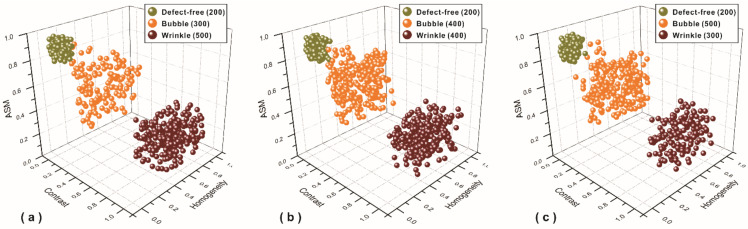
Plot of KMC algorithm classification for the three conditions with proportions of (**a**) 2:3:5, (**b**) 2:4:4, and (**c**) 2:5:3.

Moreover, a total of 1000 samples based on the same three conditions (defect-free, bubble, and wrinkle) with proportions of 2:3:5, 2:4:4, and 2:5:3, respectively, were used to verify the recognition rate. The plots for each proportion are shown in Figure 13. The initial and final center points of the clustering results are shown in Table 4 and Table 5 respectively. The overall recognition accuracy (IE) listed in Table 6 is 94.41%, where TN, TB, and TW are the number of defect-free, bubble and wrinkle samples, RN, RB, and RW are the number of correctly recognized data elements, IN, IB, and IW are the recognition accuracy, and IM is their average.

**Figure 14:**
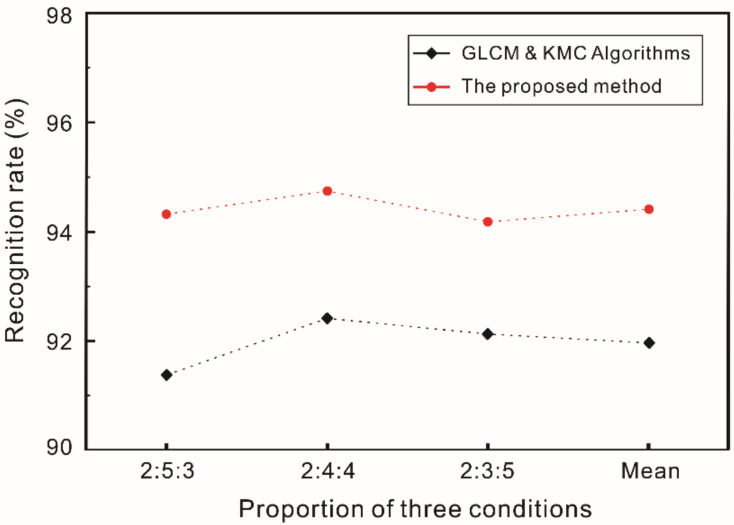
Diagram of the algorithm comparison.

**Figure 15:**
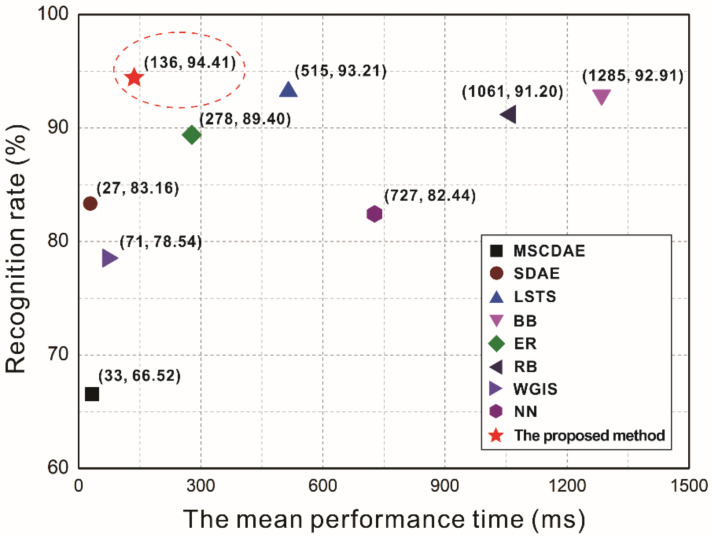
Comparison of different recognition methods (MSCDAE [40], SDAE [41], LSTS [30], BB [23], ER [25], RB [24], WGIS [26], NN [42]).

When texture feature extraction and defect type inspection was performed only by GLCM and KMC the recognition accuracy was 91.96%, as shown in Table 7. At the same time, the image processing time is also slower than that of the above method, as shown in Table 8. In contrast with general image preprocessing and the GLCM algorithm, this paper further refines the processing of texture features. Therefore, the recognition accuracy can be significantly improved in different proportions, as shown in Figure 14. Furthermore, the proposed method was compared with several existing computer vision and machine learning methods based on fiber recognition, with the results shown in Figure 15. Despite the different data sets employed by the above methods, their final purpose is ultimately to effectively recognize fiber morphologies and defect types. The proposed method can describe the morphological characteristics of defects in carbon fiber prepreg more comprehensively and more simply, and shows a remarkable improvement recognition accuracy; the results show that the proposed methodology is competitive or better than these traditional techniques. The three kinds of surface morphologies (defect-free, bubble, and wrinkle) in carbon fiber prepreg were all recognized effectively.

## 5. Conclusions

Here, we have proposed a novel defect inspection method to improve the efficiency pf automatic online detection of defects in carbon fiber plain-woven prepregs, which can help in developing an automatic laying-up process in their production. The proposed method compresses a plain-woven prepreg image while reserving additional texture features, which are calculated using GLCM. These features can be regarded as a three-dimensional array and used as the input of the KMC algorithm, which is then applied to define the defect types and realize defect inspection. It should be noted that the performance of the proposed method was compared to several different inspection methods, it showed significant improvement; the recognition rate and performance time of the proposed method reached 94.41% and 136 ms, respectively. Moreover, this proposed method could be used for surface defect identification in other woven patterns, such as twill and satin.

## Figures and Tables

**Table 1:** The co-occurrence matrices of two conditions [34].

Function	Position	Conditions
*M* (*i*, *j*, *d*, 45*°*)	*R_RD_*(*d*): (*x*_1_ − *x*_2_= *d*, *y*_1_ − *y*_2_= −*d*) or (*x*_1_ − *x*_2_= -*d*, *y*_1_ − *y*_2_= *d*)	{*R_RD_*(*d*), *f*(*x*_1_, *y*_1_) = *i*, *f*(*x*_2_, *y*_2_) = *j*}
*M* (*i*, *j*, *d*, 135*°*)	*R_LD_*(*d*): (*x*_1_ − *x*_2_= *d*, *y*_1_ − *y*_2_= *d*) or (*x*_1_ − *x*_2_= -*d*, *y*_1_ − *y*_2_= −*d*)	{*R_LD_*(*d*), *f*(*x*_1_, *y*_1_) = *i*, *f*(*x*_2_, *y*_2_) = *j*}

**Table 2:** Performance evaluation metrics for vertical and horizontal steerable filtered images.

Image Size	Direction	ACC	TPR	FPR	PPV	NPV
10 × 10	Vertical	82.27	78.13	13.31	86.27	78.74
	Horizontal	72.89	72.24	26.39	74.71	71.07
Full-size	Vertical	77.33	69.57	19.29	81.43	73.36
	Horizontal	63.58	36.49	47.33	90.01	56.19

**Table 3:** The mean value of textural features.

Specimens	Contrast	Homogeneity	Angular Second Moment
Defect-free	0.032	0.022	0.960
Bubble	0.272	0.242	0.680
Wrinkle	0.839	0.830	0.177

**Table 4:** The initial cluster center of three surface morphologies with different proportions.

Defect-Free: Bubble: Wrinkle	Defect-Free Cluster Center Coordinates	Bubble Cluster Center Coordinates	Wrinkle Cluster Center Coordinates
2:3:5	(0.0352, 0.0225, 0.9556)	(0.2746, 0.2456, 0.6734)	(0.8345, 0.9934, 0.1579)
2:4:4	(0.0352, 0.0225, 0.9556)	(0.2746, 0.2456, 0.6734)	(0.8345, 0.9934, 0.1579)
2:5:3	(0.0352, 0.0225, 0.9556)	(0.2746, 0.2456, 0.6734)	(0.8345, 0.9934, 0.1579)

**Table 5:** The final cluster center of three surface morphologies with different proportions.

Defect-Free: Bubble: Wrinkle	Defect-Free Cluster Center Coordinates	Bubble Cluster Center Coordinates	Wrinkle Cluster Center Coordinates
2:3:5	(0.0321, 0.0203, 0.9596)	(0.2696, 0.2425, 0.6759)	(0.8389, 0.8297, 0.1779)
2:4:4	(0.0322, 0.0217, 0.9598)	(0.2719, 0.2427, 0.6799)	(0.8393, 0.8304, 0.1773)
2:5:3	(0.0319, 0.0221, 0.9603)	(0.2734, 0.2408, 0.6811)	(0.8432, 0.8314, 0.1763)

**Table 6:** The recognition accuracy of the proposed method in this paper.

Proportion	T_N_/T_B_/T_W_	R_N_/R_B_/R_W_	I_N_/%	I_B_/%	I_W_/%	I_M_/%	I_E_/%
2:3:5	200:300:500	188:283:471	94.00	94.33	94.20	94.18	94.41
2:4:4	200:400:400	189:379:380	94.50	94.75	95.00	94.75	
2:5:3	200:500:300	189:469:284	94.50	93.80	94.67	94.32	

**Table 7:** The recognition accuracy of the GLCM algorithm.

Data Set	Proportion	Recognition Accuracy (%)	Average Value (%)
1	2:3:5	92.12	91.96
2	2:4:4	92.41	
3	2:5:3	91.37	

**Table 8:** The effect of image compression on performance time.

Method	GLCM & KMC Method	The Proposed Method of This Article
Each testing time (ms)	472	136
Total testing time (min)	7.87	2.27

## Data Availability

The data presented in this study are available on request from the corresponding author.

## Nomenclature

***O*** *_org_*	original image matrix
w	image width (px)
*h*	image height (px)
*n_x_*	the numbers of warps
*n_y_*	the numbers of wefts
***W** ^i^*	weft-block matrix
$\bar{t}$	binary threshold value
***O*** *_bnr_*	binary matrix
***C** ^il^*	crossing-block matrix
***F***	output matrix
***M*** *_ij_*	co-occurrence matrix
C	contrast
H	homogeneity
E	angular second moment

## Appendix A

Let us recall that when the prepreg images captured in the process of automatic placement need to be enhanced by three preprocessing methods: (1) histogram equalization; (2) gray-level morphology; (3) steerable filters; (4) Performance evaluation metrics; (5) Gray level co-occurrence matrix. The specific algorithm implementation of these methods is as follows.

(1) *Histogram equalization:* Firstly, the global image is equalized directly. Suppose that the histogram distribution of the captured image *A* is *H_A_(D)*. The monotone nonlinear mapping is used to change image *A* into image *B*, that is, function transformation ***f*** is applied to each pixel in image *A*, and the histogram of image *B* is obtained as *H_B_(D)*.

The whole process can be understood as changing all the *D_A_* in image *A* into *D_B_* as follows:

(A1) $$\int_{D_{A}}^{D_{A}+\Delta D_{A}}H_{A}\left(D\right)dD=\int_{D_{B}}^{D_{B}+\Delta D_{B}}H_{B}\left(D\right)dD$$

In order to achieve histogram equalization, the following are in particular:

(A2) $$\int_{0}^{D_{A}}H_{A}\left(D\right)dD=\int_{0}^{D_{B}}H_{B}\left(D\right)dD$$

Because the target is histogram uniformly distributed, we obtain *H_B_(D)* = *A_0_*/*L*, where *A_0_* is the number of pixels, and *L* is the grayscale depth (usually 256). And then we get:

(A3) $$\int_{0}^{D_{A}}H_{A}\left(D\right)dD=\frac{A_{0}D_{B}}{L}=\frac{A_{0}f\left(D_{A}\right)}{L}$$

Therefore, let us define ***f***:

(A4) $$f\left(D_{A}\right)=\frac{L}{A_{0}}\int_{0}^{D_{A}}H_{A}\left(D\right)dD$$

The discrete form is:

(A5) $$f\left(D_{A}\right)=\frac{L}{A_{0}}\sum_{u=0}^{D_{A}}H_{A}\left(u\right)$$

Then the histogram distribution in the local area window is used to construct the mapping function to equalize the local image area.

Finally, in order to avoid the discontinuity and excessive enhancement of the image, the interpolation method is used to accelerate the histogram equalization. Bilinear interpolation formula is as followed.

(A6) $$f\left(D\right)=\left(1-\Delta y\right)\left(\left(1-\Delta x\right)f_{ul}\left(D\right)+\Delta xf_{bl}\left(D\right)\right)+\Delta y\left(\left(1-\Delta x\right)f_{ur}\left(D\right)+\Delta xf_{br}\left(D\right)\right)$$

where *f_ul_(D)*, *f_ur_(D)*, *f_dl_(D)*, *f_dr_(D)* are the mapping values of the histogram CDF of the adjacent four windows to the central pixel, and Δ*x*, Δ*y* are the ratio of the distance between the center pixel and the window pixel and the window size.

(2) *Gray-level morphology*: The operation object of gray-level mathematical morphology is not a set, but an image function. The dilation and erosion operations of the input image *f(x, y)* with structure element *b(x, y)* are respectively defined as:

(A7)
$$\left(f⊕b\right)\left(s,t\right)=max\left\{f\left(s-x,t-y\right)+b\left(x,y\right)\left|\left(s-x,t-y\right)\in D_{f},\left(x,y\right)\in D_{b}\right.\right\}$$

(A8)
$$\left(f⊙b\right)\left(s,t\right)=min\left\{f\left(s+x,t+y\right)+b\left(x,y\right)\left|\left(s+x,t+y\right)\in D_{f},\left(x,y\right)\in D_{b}\right.\right\}$$

Opening and closing operations are respectively defined as:

(A9) $$f_{opn}=\left(f⊙b\right)⊕b$$

(A10) $$f_{cls}=\left(f⊕b\right)⊙b$$

The operations of top-hat and bottom-hat are respectively defined as:

(A11) $$f_{top}=f-f_{opn}$$

(A12) $$f_{btm}=f_{cls}-f$$

Therefore, we obtain the final gray-level morphology preprocessing formula:

(A13) $$f_{final}=f+f_{top}-f_{btm}$$

(3) *Steerable filters:* Considering the woven pattern, we use the steerable vertical filters with the following basic 2-D Gaussian function in this paper.
  (A14) $$f\left(x,y\right)=\frac{1}{2\pi \sigma_{x}\sigma_{y}}exp\left[-\left(\frac{x^{2}}{2\sigma_{x}{}^{2}}+\frac{y^{2}}{2\sigma_{y}{}^{2}}\right)\right]$$
  where *σ_x_* and *σ_y_* are the standard deviation in the x and y directions, respectively.

Rotation matrix (Equation (A15)) is used to rotate the axis in accordance with a necessary angle *θ* into the function.

(A15) $$\left[\begin{array}{c} X\\ Y \end{array}\right]=\left[\begin{array}{cc} \cos\theta & \sin\theta \\ -\sin\theta & \cos\theta \end{array}\right]\left[\begin{array}{c} x\\ y \end{array}\right]$$

Therefore, the steerable vertical filter algorithm is derived from Equation (A16). After defining appropriate parameters of *σ_x_*, *σ_y_* and *θ*, the filter is convolved with above-mentioned morphology enhanced image.

(A16) $$f\left(x,y\right)=\frac{1}{2\pi \sigma_{x}\sigma_{y}}exp\times \left[-\frac{{\left(x\cos\theta +y\sin\theta \right)}^{2}}{2\sigma_{x}{}^{2}}-\frac{{\left(-x\sin\theta +y\cos\theta \right)}^{2}}{2\sigma_{y}{}^{2}}\right]$$

(4) *Performance evaluation metrics*: First, the numerical comparisons between steerable filtered images (binary images after filter) and template images are conducted in a pixel-by-pixel manner. Specifically, both pixel values in the filtered and template images are 255 as true positive (TP), while 0 as true negative (TN). The pixel value in the filtered image is 255 and that of the defect manual-labeled image is 0 as false positive (FP), while the reversed situation is false negative (FN). The following measurement metrics are used to compare the result of applying vertical and horizontal steerable filters:

(A17)
$$ACC=\frac{TP+TN}{TP+FN+FP+TN}$$

(A18)
$$TPR=\frac{TP}{TP+FN}$$

(A19)
$$FPR=\frac{FP}{FP+TN}$$

(A20)
$$PPV=\frac{TP}{TP+FP}$$

(A21)
$$NPV=\frac{TN}{TN+FN}$$

In the above formula, ACC is accuracy, TPR is true positive rate, FPR is false positive rate, PPV is positive predictive value, NPV is negative predictive value.

(5) *Gray level co-occurrence matrix*: All values of the co-occurrence matrices are given normalized treatment before the texture features are calculated. The co-occurrence matrices, *M_ij_*, is shown as Equation (A22).

(A22)
$$M_{ij}=\frac{P_{ij}}{\sum_{i=0}^{l}\sum_{j=0}^{l}P_{ij}}$$

Contrast, homogeneity and angular second moment are used to describe the carbon fiber texture features.

Contrast: The contrast reflects the sharpness of the image and the depth of the texture patterns. The pattens is deep, the image is clear, and the contrast is large. On the contrary, the patterns are shallow, the image is fuzzy, and the contrast is small.

(A23) $$C=\overset{N-1}{\underset{t=0}{\sum }}t^{2}\left\{\overset{l-1}{\underset{i=0}{\sum }}\overset{l-1}{\underset{j=0}{\sum }}P^{2}\left(i,j\right)\right\}$$

(A24) $$t=\left|i-\left.j\right|\right.$$

Homogeneity: The homogeneity measures how much the image texture changes locally. The larger the value is, the less variation of different regions of the image texture is, and the local texture is very uniform.

(A25) $$D=\overset{l-1}{\underset{i=0}{\sum }}\overset{l-1}{\underset{j=0}{\sum }}P\left(i,j\right)\frac{1}{1+{\left(i-j\right)}^{2}}$$

Angular second moment (ASM): ASM is the sum of squares of element values in the gray co-occurrence matrix. It reflects the degree of texture thickness and the degree of evenness of image gray-level distribution. When the value is larger, gray-level distribution is uniform and image is closed-grain. Otherwise, it is smaller.

(A26) $$E=\overset{l-1}{\underset{i=0}{\sum }}\overset{l-1}{\underset{j=0}{\sum }}P^{2}\left(i,j,d,\theta \right)$$
